# Case Report: Composite pheochromocytoma with ganglioneuroma component: A report of three cases

**DOI:** 10.3389/fendo.2022.903085

**Published:** 2022-09-14

**Authors:** Paula B. Araujo, Mirna S. Carvallo, Ana P. Vidal, João B. Nascimento, Julia M. Wo, Erika O. Naliato, Silvio H. Cunha Neto, Flavia L. Conceição, Rosita Fontes, Vinicius V. de Lima, Denise P. Carvalho, Paula Soares, Jorge Lima, Delmar M. Lourenço, Alice Helena D. Violante

**Affiliations:** ^1^ Medical Board of Clinical Analysis Department, Dasa, Rio de Janeiro, Brazil; ^2^ Medical School, Endocrine Service, Universidade Federal do Rio de Janeiro, Rio de Janeiro, Brazil; ^3^ Medical School, Universidade Federal do Rio de Janeiro, Rio de Janeiro, Brazil; ^4^ Pathology Service Clementino Fraga Filho University Hospital, Universidade Federal do Rio de Janeiro, Rio de Janeiro, Brazil; ^5^ Ricardo Castilho Center of Studies Teresopolis Medical Association, Teresopolis, Rio de Janeiro, Brazil; ^6^ Endocrine Surgery Service, Hospital Universitario Clementino Fraga Filho, Faculdade de Medicina, Universidade Federal do Rio de Janeiro, Rio de Janeiro, Brazil; ^7^ Medical School, Endocrinology Unit, Hospital Universitario Clementino Fraga Filho, Faculdade de Medicina, Universidade Federal do Rio de Janeiro, Rio de Janeiro, Brazil; ^8^ Laboratorio de Fisiologia Endocrina Doris Rosenthal, Instituto de Biofísica, Universidade Federal do Rio de Janeiro, Rio de Janeiro, Brazil; ^9^ Institute of Molecular Pathology and Immunology of the University of Porto (IPATIMUP), Porto, Portugal; ^10^ Institute of Research and Innovation in Health of the University do Porto, Porto, Portugal; ^11^ Department of Pathology, Faculty of Medicine, University of Porto, Porto, Portugal; ^12^ Department of Medicine, Faculty of Medicine, University of Porto, Porto, Portugal; ^13^ Endocrine Genetic Unit, Endocrinology Division, Hospital das Clínicas, University of Sao Paulo School of Medicine, University of Sao Paulo, São Paulo, Brazil; ^14^ Endocrine Oncology Division, Institute of Cancer of the State of Sao Paulo, University of Sao Paulo School of Medicine, University of Sao Paulo, São Paulo, Brazil

**Keywords:** pheocromocytoma, ganglioneuroma, composite pheocromocytoma, molecular study, pathology, paraganglioma

## Abstract

Composite pheochromocytoma (CP) is a very rare tumor originating from neural crest cells, predominantly composed of pheochromocytoma (PCC), a chromaffin cell tumor arising in adrenal medulla, and ganglioneuroma, a tumor derived from autonomic ganglion cells of the nervous system. Moreover, CP may be present in the hereditary syndromes of which pheochromocytoma is part. Literature offers scarce data on this subject, and particularly about its biological behavior, clinical evolution, and molecular profile. We report the phenotype and outcome of three cases of CP (PCC and ganglioneuroma components), followed up at the Endocrine Service of the Clementino Fraga Filho University Hospital, Federal University of Rio de Janeiro, UFRJ, Rio de Janeiro, Brazil. Two nonsyndromic patients (cases 1 and 2) were negative to germline mutations in genes *VHL*, *SDHB*, *SDHC*, *SDHD*, *SDHAF2*, *TMEM127*, and *MAX*, while the third case (case 3) had clinical diagnosis of neurofibromatosis syndrome. Cases 1, 2, and 3 were diagnosed at 29, 39, and 47 years old, respectively, and were followed up for 3, 17, and 9 years without no CP recurrence. All cases had apparent symptoms of catecholaminergic excess secreted by PCC. Ganglioneuroma, the neurogenic component present in all three cases, had a percentage representation ranging from 5% to 15%. Tumors were unilateral and large, measuring 7.0 cm × 6.0 cm × 6.0 cm, 6.0 cm × 4.0 cm × 3.2 cm, and 7.5 cm × 6.0 cm × 4.5 cm, respectively. All cases underwent adrenalectomy with no recurrence, metastasis, or development of contralateral tumor during follow-up. Genetic testing has been scarcely offered to CP cases. However, a similar frequency of genetic background is found when compared with classic PCC, mainly by the overrepresentation of NF1 cases in the CP subset. By literature review, we identified a notorious increase in cases reported with CP in the last decade, especially in the last 3 years, indicating a recent improvement in the diagnosis of this rare disorder in clinical practice.

## Introduction

Composite pheochromocytoma (CP) is a very rare clinical condition with almost a hundred cases reported in the medical literature and constitutes less than 3% of all adrenal gland neoplasms and sympathoadrenal pheochromocytomas. CP is characterized by the coexistence of pheochromocytoma (PCC) or paraganglioma (PGL) with other neurogenic tumors, such as ganglioneuroma, ganglioneuroblastoma, neuroblastoma, schwannomas, malign peripheral nerve sheath tumors, such as sustentaculoma, rhabdomyosarcoma, and neuroendocrine carcinoma. Ganglioneuroma is the most common tumor among neurogenic tumors in the context of CP, accounting for 75% of the cases reported (1–8).

Of note, most patients with CP present clinical manifestations of catecholamine excess (three-quarters), with no distinctive clinical or radiological feature from usual PCC or PGL. Thus, the diagnosis of CP is based only on pathological findings (1, 9).

Of value, the diagnosis of CP should be warranted if at least 5% of one of the endocrine or neural components is documented, as it is not uncommon for some mature ganglion cells or sparsely dispersed Schwannian-like stroma to occur in typical PCC or PGL (8). The prompt and accurate diagnosis of neural and endocrine components may be important, mainly if one of them is associated with undifferentiated or poorly differentiated tumors compromising the prognosis of these patients as, for example, presence of neuroendocrine carcinoma or spindle cell sarcoma (8, 10).

Pheochromocytoma may be considered the neoplasia of higher genetic variability known as more than 35% of the patients harbor a germline mutation found in one of at least 16 possible genes, and it is amplified if somatic mutations were considered (11, 12). In contrast, only a few studies have supported genetic testing to CP and, thus, the molecular profile of these cases is not fully known, except by an apparent overrepresentation of neurofibromatosis (NF1) (4, 7).

In the present study, we report the phenotypic features and outcome of three cases documented with CP by coexistence of PCC and ganglioneuroma. Furthermore, investigation of germline allelic variants, directed by phenotype, was applied to two of them with sporadic and nonsyndromic presentation. In addition, an extensive review of the current literature of previously reported cases, an apparently peculiar genetic profile, and suspected underdiagnosis of this rare condition are presented and discussed.

## Materials and methods

### Clinicopathologic information

After giving informed consent, as established in the approval by the Ethics Committee of our Institution, three consecutive patients with CP, diagnosed between 2004 and 2018, were enrolled in this study. Patient outcomes on follow-up and clinicopathologic information, including serum catecholamine levels and disease associations, were obtained from the medical record.

### Pathology and immunohistochemistry

The surgical specimens obtained from the adrenalectomy of the three enrolled patients were fixed in formalin and embedded in paraffin (FFPE), and sections were stained with hematoxylin–eosin (HE). Tissue samples representative of the different macroscopic aspects identified in the large adrenal tumors of cases 1 and 3 were collected and included. In turn, the surgical specimen from case 2 was entirely included.

The pheochromocytoma of the adrenal gland scaled score (PASS score) was established for all the cases. Representative tissue blocks were selected for each case. Immunohistochemical (IHC) analysis was done with the following antibodies: chromogranin A (CgA), clone 5H7, obtained from Leica Biosystems (dilution: 1:1,000); neurofilament (NF), clone 2F11, from Dako Cytomation (1:3,000); S100, from Dako Cytomation (1:5,000); and Ki-67, clone SP6, from Spring Bioscience (1:300). Five areas of greater Ki-67 expression intensity (hot spots) were selected and counted to define label index.

### Genetic analyses

DNA samples from all patients were obtained from peripheral blood using the modified Miller method at the Carlos Chagas Filho Biophysics Institute (UFRJ). Subsequently, the material was prepared at the Institute of Molecular Pathology and Immunology of the University of Porto (IPATIMUP) for sequencing of the following genes: *VHL*, *SDHB*, *SDHC*, *SDHD*, *SDHAF2*, *TMEM127*, and *MAX*. The polymerase chain reaction (PCR) was used to amplify the exons and exon–intron boundaries of the mentioned genes. The PCR reactions were carried out using genomic DNA (100–500 ng), 200 µM of each deoxynucleotide (dNTP), 10–30 pmol of each primer, 2.5 U of Taq DNA polymerase enzyme, and 5 μl of the buffer supplied by the manufacturer (Amersham Pharmacia, Uppsala, Sweden) and H_2_0 MiliQ to be completed for a final volume of 25 µl.

The amplification protocol consisted of denaturation at 94°C for 5 min, followed by 35 to 40 cycles (with hybridization temperature according to oligonucleotides) of 94°C for 40 s, annealing (55°C–60°C) for 40 s and 72°C for 50 s, followed by a final extension cycle of 72°C for 7 min. The amplified fragments were subjected to electrophoresis on a 2% agarose gel stained with ethidium bromide (0.5 µg/ml) (Invitrogen™ Life Technologies, Carlsbad, CA, USA). PCR products were purified using the QIAquick PCR purification Kit (Qiagen Str. 1, 40724 Hilden, Germany) according to the manufacturer’s recommendations. Sequencing reactions were performed with the ABI Prism BigDye Terminator Kit (Life Technologies, Carlsbad) and the fragments were run in an ABI prism 3,100 and 3,500 xL Genetic Analyser (Life Technologies, Carlsbad).

In addition to the coding region of *SDHB*, we have also used a primer pair (forward: AGCGCCAATTGTGGAAATAG; reverse: GCCTGAGGCAGATAGTAGGG) for specifically detecting the previously described *SDHB* 15678bp deletion (c.1-10413_73-3866del; g.17043962_17059585del) that removes the promoter region and exon 1 of this gene (4).

### Case reports

#### Case 1

A 29-year-old woman presented with abdominal pain, asthenia, and diarrhea. She had no signs of arterial hypertension, despite extensive investigation. Blood tests revealed increased levels of free plasmatic epinephrine and norepinephrine (**Table 1**). Abdominal computed tomography showed an expansive and well-delimited lesion with heterogeneous enhancement by means of contrast, located in the topography of the right adrenal gland, measuring 7.0 cm × 7.4 cm × 6.6 cm, contiguous to the right hepatic lobe and the superior renal pole, without plans to cleave with the inferior vena cava. Morphology and dimensions of the left adrenal gland were normal (**Table 1**). With preoperative alpha-adrenergic blocker, patient was submitted to open right adrenalectomy without postoperative complications. A complete remission of symptoms and normal hormonal measurements were noticed after surgery, with no recurrence during 3 years of follow-up.

**Table 1:** Genetic, radiological, hormonal, and clinical profiles and outcomes of three women with composite PCC.

Cases/sex	Age at diagnosis/current age (years)	Follow-up (years)^a^/recurrence	Clinical features	Hormonal hypersecretion	Adrenalectomy (L, left; R, right)	Radiologic features (L, left; R, right)	Molecular analysis^b^
1/F	29/31	3/No	Abdominal pain; asthenia; diarrhea; no hypertension	Free plasmatic epinephrine and norepinephrine	Open (R)	R, 7.0 × 7.4 × 6.6 cm L, normal	Negative
2/F	39/56	17/No	Hypertension, sweating, weight loss	Urinary epinephrine and norepinephrine	Laparoscopic (L)	L, 3.9 cm R, normal	Negative
3/F	47/55	8/No	Hypertension; tachycardia; sweating; low left back pain; type 2 diabetes; NF1	NA	Laparoscopic (L)	L, 5.6 × 5.2 × 6.0 cm R, normal	–^c^

PCC, pheochromocytoma; F, female; NF1, neurofibromatosis type 1; NA, not available. ^a^No evidence of new PCC or even paraganglioma or any tumor during follow-up. ^b^The following genes were studied in cases 1 and 2: VHL, SDHB, SDHC, SDHD, SDHAF2, TMEM127, and MAX. ^c^Genetic analysis was not performed as the patient had an irrefutable clinical diagnosis of NF1.

#### Case 2

A 39-year-old woman presented with severe paroxysmal systemic arterial hypertension, sweating, and progressive weight loss. Hormonal evaluation revealed high values of urinary epinephrine and norepinephrine (**Table 1**). Magnetic resonance image showed a lesion of 3.9 cm in the left adrenal gland with normal morphology and dimensions of the right adrenal (**Table 1**). She also received alpha-adrenergic blockade before laparoscopic adrenalectomy without any perioperative relevant intercurrences. A complete remission of symptoms was noticed after adrenalectomy, with no recurrence after 17 years of follow-up.

#### Case 3

A 47-year-old woman, with type 2 diabetes mellitus, systemic arterial hypertension, and a previous clinical diagnosis of neurofibromatosis type 1 (NF1), presented with persistent left low back pain, tachycardia, and sweating (**Table 1**). Computed tomography showed a nodular formation, with soft tissue density, heterogeneous, with intervening hypodense areas and tenuous parietal calcifications, with early heterogeneous contrast enhancement, measuring 5.6 cm × 5.2 cm × 6.0 cm, located in the left adrenal, suggesting pheochromocytoma. The morphology of the contralateral adrenal was normal (**Table 1**). A baseline hormonal profile was not performed, and the patient was referred to our service only after laparoscopic left adrenalectomy. Also, there was no previous clinical history of use of preoperative medication preparation (alpha blockade) or of early peri- and postoperative complications. Also, there was no recurrence of CP during 9 years of follow-up (**Table 1**).

### Pathological features

**Figure 1:**
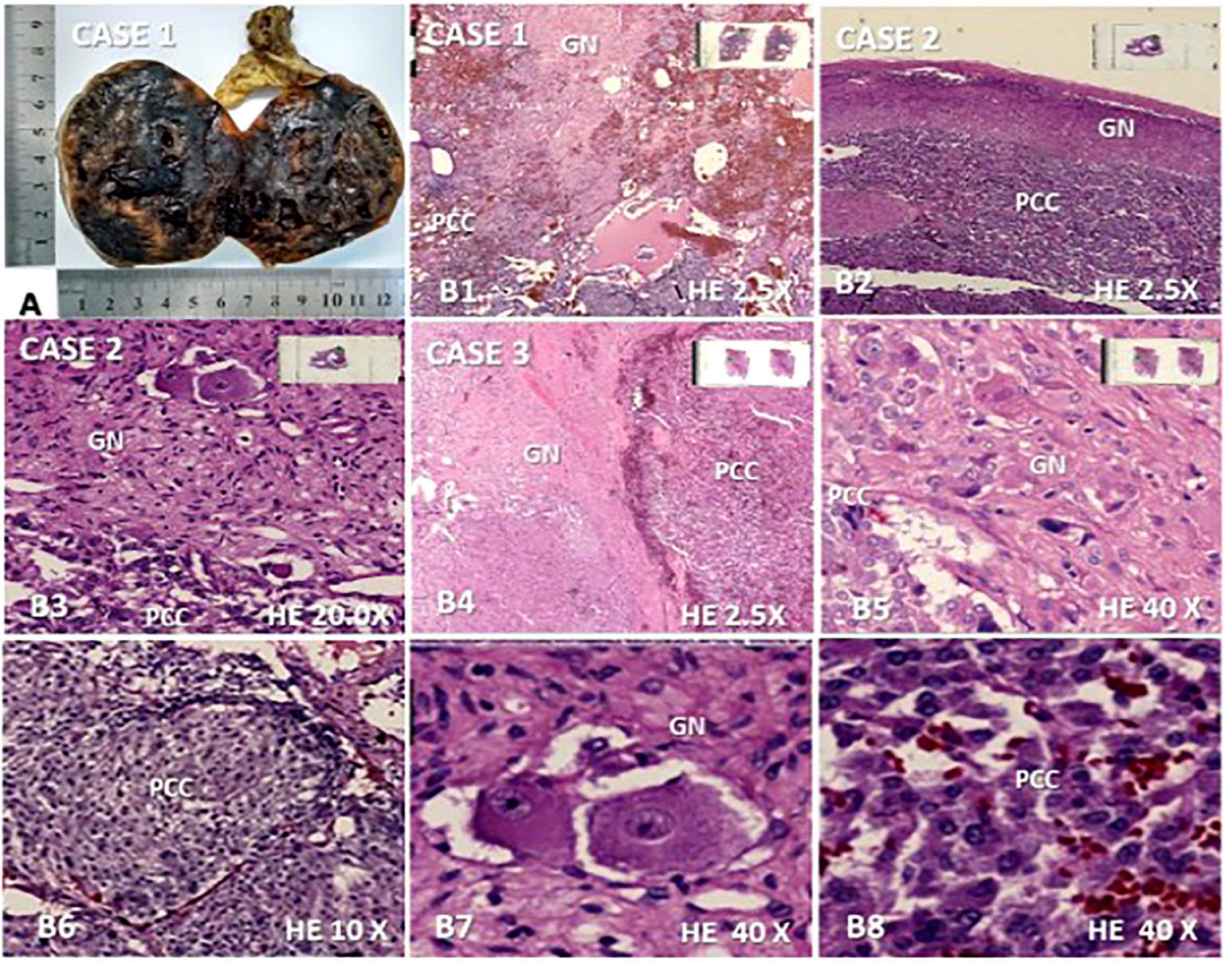
Macroscopic and microscopic features found in the three composite pheochromocytoma (CP) cases with ganglioneuroma as a neurogenic component. **(A)** Opened surgical specimen of case 1 measuring 7.0 ×6,0 × 6.0 cm; **(B1**–**B8)**, microscopy (hematoxylin-eosin) revealing the presence of both components of CP in cases 1, 2 and 3: **(B1)**. ganglioneuroma (GN) interspersed between pheochromocytoma (PCC) and cystic/hemorrhagic areas (2.5×); **(B2)**. ganglioneuroma presenting as a peripheral ridge adjacent to the chromatin tumor in case 2 (2.5×); **(B3)**. GN dispersed in most part of the figure with clear margin of frontier with PC component located in inferior region (20×); **(B4**, **B5)**. well-delimited neurogenic component of the CP of the case 3 is shown on the left side **(B4)** (2.5×) and in most part of the figure **(B5)** (40×) while PCC is seen, on the right side **(B4)** (2.5) and in the left inferior region **(B5)** (40×); **(B6)**. A nodular area of PC is represented (10×); **(B7)**. Ganglion cells dispersed in Schwan stroma; **(B8)**. Chromaffin cells network of PCC.

A histopathological study of the three cases revealed a composite PCC whose neurogenic component was a ganglioneuroma in all of them (**Figure 1**). The percentage representation of ganglioneuroma component in the anatomic specimen was 15%, 10%, and 5% in cases 1, 2, and 3, respectively. This neurogenic component was basically present in the middle of cystic and hemorrhagic areas of the tumor in cases 1 and 3 or like a peripheral ridge in the tumor of case 2 (**Figure 1**). Cases 1 and 2 had Ki-67 of <3% and case 3 had >3%. PASS score was 7, 3, and 11 in cases 1, 2, and 3 (**Table 2**), respectively.

**Table 2:** Pathological features of three cases with composite PCC.

Cases	Age at the adrenalectomy	Pathological features of composite PCC^a, b^			
		Weight (g) [(size (cm)]	PASS	Ki-67	Neurogenic component (%)
1	29	120 7.0 × 6.0 × 6.0	7/20	<3%	Ganglioneuroma (15%)^c^
2	39	50 6.0 × 4.0 × 3.2	3/20	<3%	Ganglioneuroma (10%)^d^
3	47	90 7.5 × 6.0 × 4.5	11/20	>3%	Ganglioneuroma (5%)^c^

PCC, pheochromocytoma. ^a^All cases had positive immunohistochemistry (IHC) to chromogranin (CgA) at the cytoplasm of PCC cells and to neurofilament (NF) in the cytoplasm of neuronal cells (aggregated or diffuse presentation). ^b^IHC of S100 protein was positive in sustentacular cells of PCC as well as in Schwann cells from ganglioneuroma. ^c^It was present in the middle of cystic and hemorrhagic areas of the tumor. ^d^It was present as a peripheral ridge in the tumor of case 2.

**Figure 2:**
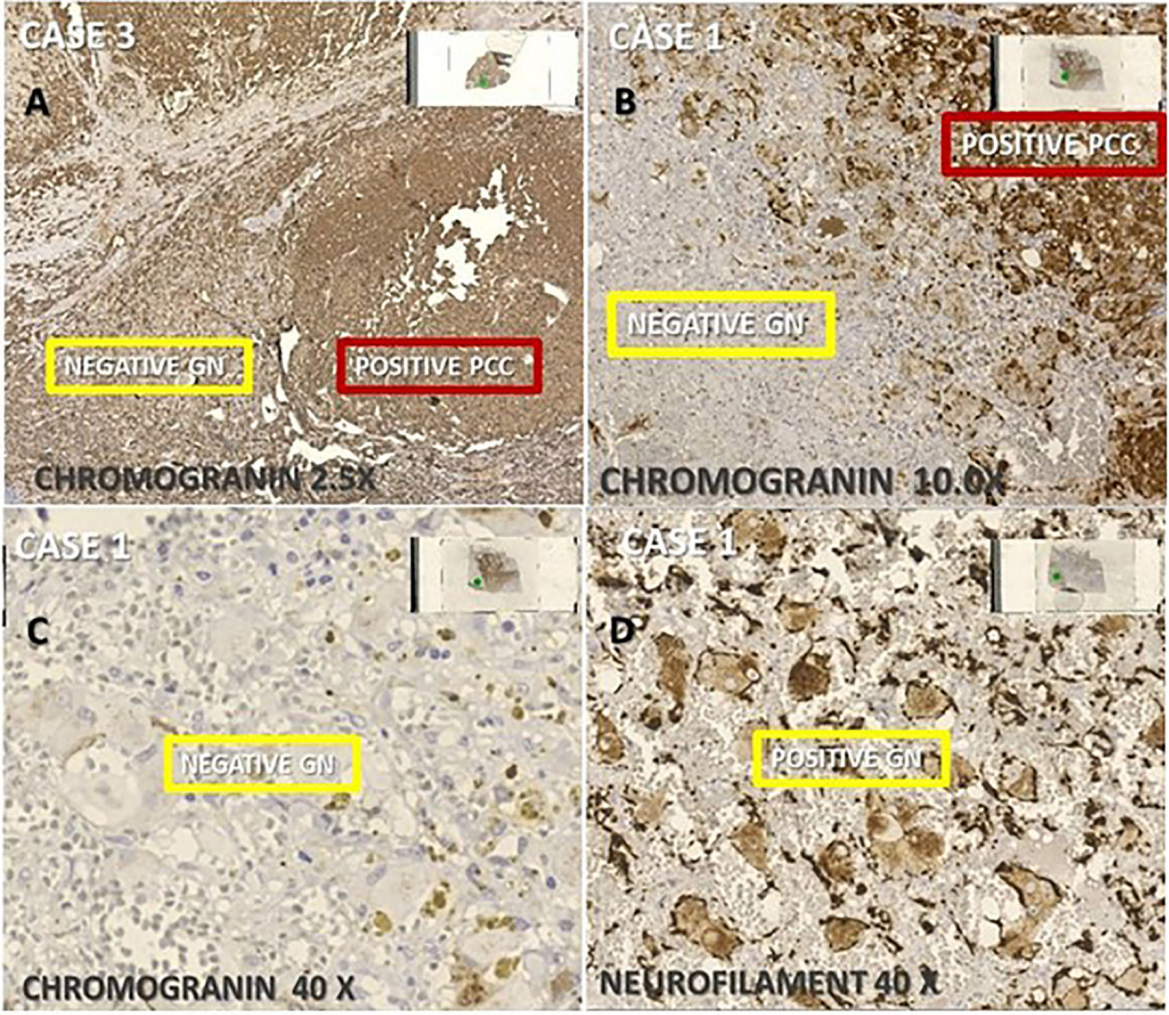
Immunohistochemistry (IHQ) of the three composite pheochromocytoma (CP) cases associated with ganglioneuroma, **(A)** ×2.5 and **(B)** ×10. IHQ was positive for chromogranin in pheochromocytoma (PCC) and negative ganglioneuroma (GN); **(C)** negative expression of chromogranin in GN; and **(D)** positive IHQ for neurofilament in GN.

In all cases, by IHC, chromogranin A (CgA) highlighted the cytoplasm of PCC cells and aggregated or diffuse neuronal cells showed cytoplasmatic positivity of neurofilament (NF) (**Figure 2**). In turn, IHC to S100 protein had strong positivity in sustentacular cells of PCC as in Schwann cells from ganglioneuroma. Thus, the pathological diagnosis of CP in all three cases was defined by a combination of microscopic findings from sections stained by HE and IHC features.

#### Case 1

The right adrenal tumoral specimen measured 7.0 cm × 7.4 cm × 6.6 cm, weighed 120 g, and had a thick capsule and a smooth brown surface. Neoplasia was composed by large cells with focal nuclear pleomorphism and nucleoli with vesicular chromatin, <3 mitosis/10 high power fields (HPF), without atypical mitosis or necrosis or angioinvasion or extra capsular extension.

#### Case 2

The tumor size was 6.0 cm × 4.0 cm × 3.2 cm, weighed 50 g, and had fibroelastic consistence and a brownish color. Microscopy revealed a lobular arrangement neoplasia with pleomorphic cells of poorly delimited granular cytoplasm, clear nuclei, and predominantly eosinophilic nucleoli.

#### Case 3

The tumoral mass measured 7.5 cm × 6.0 cm × 4.5 cm, weighed 90 g, with a well-defined lobular arrangement composed of cells with mild or moderate atypia and a rare anaplasia focus, with angioinvasion focus and extracapsular extension area to adjacent adipose tissue.

### Genetic analysis

Genetic analysis was negative for germline pathogenic variants in all genes studied (*VHL*, *SDHB*, *SDHC*, *SDHD*, *SDHAF2*, *TMEM127*, and *MAX*) of the two nonsyndromic cases (cases 1 and 2) (**Table 1**). Benign/likely benign variants were identified in *TMEN127* gene of these cases: case 1, silent variant c.621G>A p.(Ala207Ala; dbSNP:rs3852673); and case 2, missense variant c.268G>A p.(Val90Met; dbSNP: rs121908823). Case 3 was exempted from genetic analysis due to the irrefutable clinical diagnosis of NF1 syndrome.

## Discussion

In the present study, we reported the phenotype and outcome of three cases diagnosed with composite pheochromocytoma (CP) between 2004 and 2018. All of them had an association of pheochromocytoma (PCC) and ganglioneuroma.

Our cases represent the most frequently reported phenotype of CP, with ganglioneuroma being the main neurogenic component in 65%–71% of the cases and PCC being the dominant endocrine component (4, 7) in over 70% of the cases reported (6).

While PCCs/PGs are tumors that originate from the chromaffin cells, ganglioneuroma represents the mature spectrum of tumors from autonomic ganglion cells or their precursors. Embryologically, both chromaffin and ganglion cells are derived from neural crest cells. Any disturbance in the migration or development of the neural crest may result in the development of composite tumors (2, 4). The malignant potential of the PCC component is extremely rare in the composite tumors associated with ganglioneuroma, occurring in only 3% of the cases (4). In fact, these tumors usually have a benign behavior with no recurrence or metastatic disease, as seen in our cases (6). In turn, most cases with malign disease are represented by neurogenic tumors arising of immature ganglion cells as ganglioneuroblastoma, neuroblastoma, malignant peripheral nerve sheath tumor, or neuroendocrine carcinoma (2, 4, 6).

Clinical presentation depends on whether the tumor is functional or nonfunctional, although the majority of cases present as functional at diagnosis (4, 13). Paroxysmal or sustained arterial hypertension appears at diagnosis with a frequency varying between 48% and 72%, and headaches, tachycardia, and sweating are present in 50% of CP cases (2, 13). In our report, two out of three patients presented arterial hypertension. However, a recent review documented arterial hypertension in one-quarter of CP cases (18/74) (7). Thus, one of our cases had PCC discovered incidentally, as seen in the case reported by Rao et al., which also had CP with elements of both PCC and ganglioneuroma (2).

The age at the time of diagnosis of our three cases was 29, 39, and 47 years old, respectively, which is consistent with the literature, where the majority of cases are diagnosed between the ages of 40 and 60 (4, 6, 7). The youngest and oldest cases diagnosed with CP were 4 and 86 years old, respectively (7). The frequency of CP was mildly higher in women (57% vs. 43%) in the 90 CP cases reported (7). Our presented three cases were all women.

CPs are mostly unilateral, as seen in our patients who presented unilateral lesions measuring between 6.0 and 7.5 cm in greatest diameter. In fact, bilateral PCC occurred in only 5% of the 56 cases with CP. These three bilateral PCCs all had genetic syndrome, with two having NF1 and the third having MEN2A (7). Our NF1 case did not develop contralateral PCC during 8 years of follow-up.

Exceptionally, PGL may be the tumor arising from chromaffin cells in CP as recently documented in a case with *MAX* germline mutation and retroperitoneal PGL diagnosed at 20 years old followed by unilateral multifocal PCC at 28 years old (14). PGL in CP was reported in less than 20 cases so far (15).

Immunohistochemically, the components of CP resemble their counterparts in normal tissue or in pure tumors of the same type. Therefore, staining patterns help to identify neuroblastomata’s foci and to distinguish immature neurons from neoplastic chromaffin cells of the similar size. Schwann cells and sustentacular cells stain for S100 protein, whereas axon-like processes stain for neurofilament proteins (NFPs). PCC is composed of polygonal to spindled cells arranged in an alveolar, trabecular, or solid pattern, often with an atypical Zellballen appearance (16). Also, PCC cells contain many secretory granules and stain strongly for chromogranin A and synaptophysin, whereas neurons contain relatively sparse granules and show weak or focal staining, often in a linear or punctuate pattern corresponding to cell processes (17). Immunohistochemical markers play a central role in PCC routine diagnosis. They are essential in the definition of the cell proliferation with the Ki-67 label index, when the differential diagnosis between PCC and adrenocortical tumors is an issue, and in detecting *SDHB* mutations.

**Figure 3:**
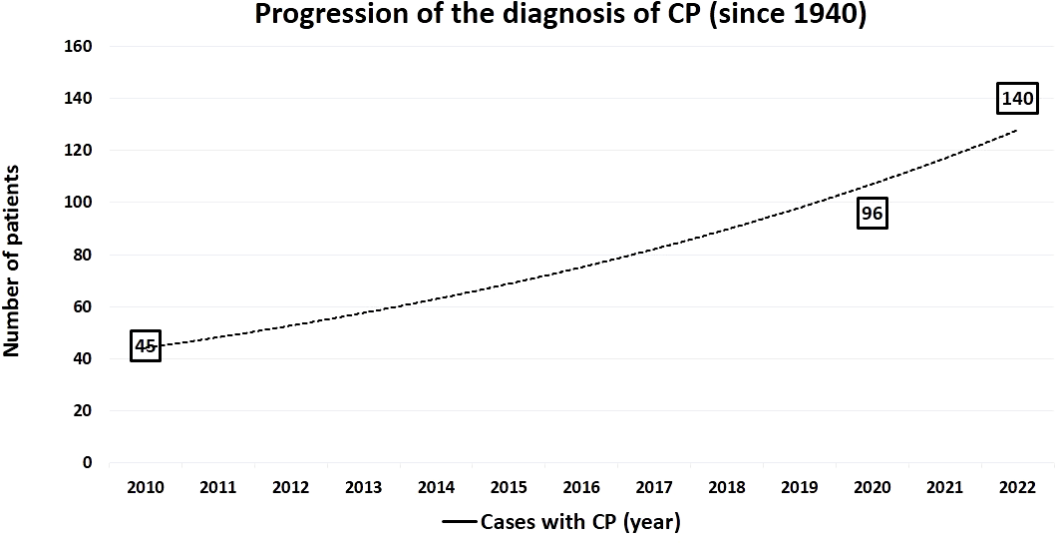
Increasing diagnosis of composite pheochromocytoma (CP) during last eight decades (1940-2022), including the three casesof the present study. CP, composite pheochromocytoma.

CP is a very rare condition, with less than 100 cases reported so far (1, 7). Since 2000, we diagnosed 20 patients with PCC and three of them were CP, which is not a negligible percentage. Interestingly, reviewing the literature, a remarkable increase in the diagnosis of CP has been reported in the last decade, mainly in the last 3 years, suggesting that the diagnosis of the disorder may have been neglected. Thus, in a long period of 70 years—between 1940 and 2010—45 CP patients have been reported so far (4, 7). In the subsequent 10 years, the number of cases reported with CP doubled (96). Of value, in a short span of 24 months—between 2020 and 2022—44 new cases were recognized, indicating that CP has been better diagnosed more recently (**Figure 3**; **Supplementary Table S1**).

It is possible that the underdiagnosis suspected of this disorder may have been associated with the absence of microscopic representation of the neurogenic component during macroscopic selection of large tumors, lack of embedding of the entire adrenal gland in paraffin or few cuts for microscopic representation of CP, increasing the risk of absence of microscopic representation of cases with a low percentage of the neurogenic component. One of our cases had only 5% of this neurogenic component, reinforcing the potential risk of underdiagnosing CP.

Also, it is relevant to note that CP have been described in the literature with a heterogeneous aspect of cystic or hemorrhagic areas and that the secondary neurogenic component is usually scant and sparse. Thus, we would like to draw attention to the importance of a careful assessment of the macroscopic aspect of tumors and the representation of areas with different aspects to ensure a proper diagnosis of the tumor. This is especially important in those cases of CP associated with neoplasms of greater biological aggressiveness such as neuroblastoma, neuroendocrine carcinoma, and malignant nerve sheath tumor (6, 7, 18). Overall, in an extensive review of the literature, Dhanasekar et al. noticed that most patients with CP had ganglioneuroma (61,6%), followed by ganglioneuroblastoma (15.1%), neuroblastoma (10.11%), schwannoma (1.1%), and others (7.7%) (7).

Despite the identification of multiple genetic causes of PCC and PGL in the last decade, around two-thirds of these tumors remain without molecular diagnosis, suggesting that other susceptibility genes could be implicated (7, 19, 20). Using a wide-genomic approach, PCC/PGL was clustered into two major groups depending on their global transcription profiles. Cluster 1 includes *VHL*, *SDHx*, *FH*-mutated tumors, and a part of the sporadic PCC/PGL. Cluster 1 was composed of tumors without clear mutations or sporadic tumors; they showed signatures of pseudohypoxia, angiogenesis, and decreased oxidoreductase response. This profile links these tumors with hypoxia-inducible factor (HIF) role, which is supported by the overexpression of HIF1α and HIF2α found in this cluster (14, 19–21). A second group (cluster 2) of PCC/PGL is related to mutations in *RET*, *NF1*, *KIF1β*, and *TMEM127* genes and to undefined tumors enriched for kinase receptor signaling pathways, translation initiation, protein synthesis, and genes involved in neural/neuroendocrine identity (20, 21).

It is recognized that CP can be associated with genetic disorders such as NF1, von Hippel–Lindau disease, and multiple endocrine neoplasia (16, 17, 21–25). In a recent systematic review, genetic syndromes were associated with CP in 28% of the patients (26/94), being: NF1 (19%), MEN2A, (4%), Von Hippel–Lindau (VHL) (2%), and watery-diarrhea hypokalemia–achlorhydria (WHDA) syndrome (2%). This prevalence was similar to that described in PCC alone (7). However, the prevalence of NF1 seems to be overrepresented in CP when compared with NF1 in large series of PCC/PGL (19% vs. 3%) (7, 25). One of our cases had NF1, reinforcing that CP may be more prevalent in this genetic syndrome.

Thus, we recommend extra attention during macroscopic analysis by the pathologist to provide microscopic representation of heterogeneous regions of the surgical specimen in PCC or PGL and, when it is possible, the full inclusion of the tumor in paraffin, especially in NF1 cases. It is possible that with this strategy, more cases with CP will be diagnosed. With this caution, immature forms of neurogenic component could eventually be revealed or excluded. This rationale is reinforced by extremely variable percentual representation of both components of CP ranging from 10% to 80% (26), suggesting that the diagnosis of some cases of CP are missed.

Reviewing the literature, only a few studies have offered genetic testing to cases with CP. (**Supplementary Table S2**). We supported genetic investigation of the germline for the main PCC-related genes. The genetic analysis of NF1 in case 3 was not performed as this patient had an irrefutable clinical diagnosis of this inherited disorder. We are not able to identify any pathogenic variants in *VHL*, *SDHB*, *SDHC*, *SDHD*, *SDHAF2*, *TMEM127*, or *MAX* genes in our two nonsyndromic CP cases. The *RET* analysis was waived in these two cases based on phenotypic features of them. In fact, MEN2A or MEN2B phenotype was absent in these cases. Also, PCC is rarely the first clinical manifestation of MEN2 and, when this occurs, is most frequently associated with *RET* mutations in the codon 634, whose patients invariably have medullary thyroid carcinoma at the ages at which they were diagnosed with PCC. Furthermore, during outcome, MTC was not diagnosed, and familial history remained negative for this syndrome. Finally, the probability to presence of a *RET* mutation in a large series of PCC is 5%. Considering the phenotype and outcome of our two nonsyndromic cases, the probability of *RET* mutation would be extremely low and, if it was present, they would be defined as anecdotical MEN2 cases (25, 27, 28).

One of the limitations of our study is that germline mutations in other genes more rarely associated with PCC/PGL could be the underlying genetic cause of our cases. Thus, the *SDHA* gene, representing 1% of all PCC cases and others very rarely associated with PCC, could not be excluded (25). As of now, only one CP case has been investigated by a genetic panel based on next-generation sequencing (NGS), and it resulted in the description of the first CP case with germline MAX mutation (14).

Of value, somatic mutation analysis was not a focus of the present paper. From our knowledge, only two CP cases had tumors studied by an extensive NGS panel (26). Thus, genes causing somatic mutations, such as *ATRX* and others, were not investigated in our cases. Of value, Chen et al. (2021) revealed that protein expression of *ATRX* and *SDHB* was normal in 18 CP cases (26). Importantly, these authors discovered that 20.0% (3/15) of the tumors studied by CP (PCC and ganglioneuroma) had *BRAF* mutations (K601E and K601N) and 46.7% (7/15) had *HRAS* mutations (Q61R, Q61L, G13R) based on genetic findings from the two cases studied by the NGS panel. These mutation frequencies were both significantly higher than those reported in ordinary PCC/PGL, suggesting that the underlying molecular mechanism of CP/PG are different from those reported in PCC/PGL alone (26). Thus, further studies focusing on the genetic causes of CP should be warranted based on the apparently higher frequency of NF1, differential expression of *ATRX*, and higher frequencies of *BRAF* and *HRAS* somatic mutation.

Very recently, exome and transcriptome analyses were conducted on a composite tumor of a 5-year old boy having as components PCC and neuroblastoma. Interestingly, most mutations (80%) were shared by all samples in both components, indicating that NB and PCC evolved from the same clone. Also, the presence of mutation and focal amplification of the *FGFR1* oncogene in both components suggests that this gene may be a primary driver of this tumor (29).

Prognostic factors of PCC include Ki-67 label index, histological pattern, cellularity, coagulative necrosis, vascular/capsular invasion, and type of catecholamine production. Ki-67 index greater than 3% indicates malignant behavior (1). Only the case presenting a phenotype compatible with NF1 presented a Ki-67 higher than 3%. However, during a long follow-up time, the patient remained asymptomatic with no evidence of recurrent PCC. Our patients with PCC and ganglioneuroma without immature components presented a good prognosis during the outcome, as previously reported (4). The presence of distant metastasis is the only criterion for malignancy in CP, which is usually derived from immature component (2, 3, 23). Considering that recurrence may occur in a few cases, long-term follow-up is required in CP (3).

Little is known about the biological potential, evolution, and genetic molecular profile of CP, although they may be associated with reported genetic syndromes, similarly to classic PCC. Indolent behavior has been described, as seen in the follow-up of our patients so far. However, there have been descriptions of metastasis associated with CP with ganglioneuroblastoma (16, 22, 23). Hence, the importance of a perfect pathological diagnosis of this association should be provided.

CP is mainly represented by the association of PCC and ganglioneuroma. Overall, patients have a favorable clinical course. However, there are cases with a malignant neurogenic component. Considering the percentage variability in the presence of both components (5%–80%), careful anatomopathologic examination is essential to avoid underdiagnosis of this rare condition. Notably, by reviewing the literature, there has been an important increase in the diagnosis of CP in the last decade, especially in the last 3 years, indicating a recent improvement in the diagnosis of this disorder in clinical practice. The prevalence of germline mutation in CP seems to be similar to that of PCC. However, genetic testing has been scarcely documented in these cases. Despite this, the genetic background of CP seems to be different from that described in regular PCC by the overrepresentation of the NF1 syndrome. More extensive genetic analysis of CP, including investigation of the germline and somatic tissues, is warranted to improve knowledge of this genetic background.

## Data availability statement

The datasets presented in this study can be found in online repositories. The names of the repository/repositories and accession number(s) can be found in the article/**Supplementary Material**.

## Ethics statement

The studies involving human participants were reviewed and approved by Comite de Etica e Pesquisa da Faculdade de Medicina e do Hospital Universitario Clementino Fraga Filho - UFRJ. The patients/participants provided their written informed consent to participate in this study.

## Author contributions

PA and MC: main authors, both contributed equally to this manuscript. APV: pathologist responsible for the study of tumors, JN and JW: data collection and figure creation; SHCN surgeon e patient`s doctor, EN, FC, RF, DC: reviewers; VL: sample collection and DNA extraction; DL: reviewer and contributor for the presentation of the manuscript format; PS and JL responsible for the molecular study; AHV: coordinator, patient’s doctor and responsible for submitting the manuscript. All authors read and approved the final manuscript.

## Funding

Sources of Research Support: JN was a recipient of a fellowship by FAPERJ (Fundação de Amparo à Pesquisa do Estado do Rio de Janeiro); DL was supported by FAPESP (Fundação de Amparo à Pesquisa do Estado de São Paulo) (grant (2016/07504-2).

## Conflict of interest

The authors declare that the research was conducted in the absence of any commercial or financial relationships that could be construed as a potential conflict of interest.

## Publisher’s note

All claims expressed in this article are solely those of the authors and do not necessarily represent those of their affiliated organizations, or those of the publisher, the editors and the reviewers. Any product that may be evaluated in this article, or claim that may be made by its manufacturer, is not guaranteed or endorsed by the publisher.
