# Supplementary material for: Case Report: Composite pheochromocytoma with ganglioneuroma component: A report of three cases
Source: Front Endocrinol (Lausanne). 2022 Sep 14;13:903085. doi: 10.3389/fendo.2022.903085 (PMC9515550; doi:10.3389/fendo.2022.903085)
Supplement: Supplementary file 1 [file Table_1.docx]

**Supplementary table 1.** Previously reported cases and new CP cases since May 2020.

| **Reference** | **Year** | **Number of cases** | **Age at the diagnosis** | **Neurogenic components** |
| --- | --- | --- | --- | --- |
| Dhanasekar et al | **2021** | 96 | 48* | Ganglioneuroma 61 (65%) Ganglioneuroblastoma 15 (16%) Neuroblastoma 10 (11%)  Schwannoma 1 (1%) |
| Arikan et al | **2021** | 1 | 54 | Ganglioneuroma |
| Chen et al | **2021** | 16 | 23-68** | Ganglioneuroma |
| Dages et al | **2021** | 20 | 62.5* | Ganglioneuroma |
| Harhar M et al | **2022** | 1 | 76 | Schwannoma |
| Pozza et al | **2020** | 1 | 20 | Ganglioneuroma |
| Tasaka K et al | **2021** | 1 | 5 | Neuroblastoma |
| Turk et al | **2022** | 1 | 45 | Spindle cell sarcoma |
| Araujo et al (Present study) | **2022** | 3 | 29-47** | Ganglioneuroma |
|  | **Total** | 140 | _ | Ganglioneuroma 99/146 (69%) |

CP, composite pheochromocytoma; *, mean age; **, minimum and maximum ages
