# Supplementary material for: Case Report: Composite pheochromocytoma with ganglioneuroma component: A report of three cases
Source: Front Endocrinol (Lausanne). 2022 Sep 14;13:903085. doi: 10.3389/fendo.2022.903085 (PMC9515550; doi:10.3389/fendo.2022.903085)
Supplement: Supplementary file 2 [file Table_2.docx]

**Supplementary table 2.** Genetic syndromes reported in CP cases (genetically investigated or not) and genetically investigated non-syndromic CP.

| **Reference (year)** | **Number of syndromic CP cases or genetically investigated** | **Phenotype** | **Genetic analysis** |
| --- | --- | --- | --- |
| Dhanasekar et al (2021) * | 25 | NF1 19/96  MEN2A 4/96  Von Hippel Lindau 2/96 | NF1: -  MEN2: p.C634R (Efared, 2017; Matias-Guiu, 1998)  / p.C634Y (Gulu, 2005)  VHL: c.5557A>G (Bernini, 2001); p.L198V (Ercolino, 2008) |
| Arikan et al (2021) | 1 | NF1 | - |
| Chen et al (2021) | 2 | Non syndromic PCC | Negative ** #; |
| Tasaka K et al (2021) | 1 | Non syndromic PCC | exome and transcriptome #; *FGFR* mutation and amplification |
| Turk et al (2022) | 1 | Non syndromic PCC | negative for *SDHB* and *RET* genes |
| Pozza et al (2020) | 1 | Phenotype suggesting genetic syndrome | NGS panel*** *MAX* germline mutation (c299G>C; p.Arg100Pro) |
| Dages et al (2021) | 1 | MEN2B | *RET* (performed but not informed) |
| Total  (Genetic syndrome) | 31 |  |  |

CP, composite pheochromocytoma; PCC, pheochromocytoma; NF1, neurofibromatosis; #, genetic analysis of the tumor; *, extensive review of the literature; **, *ATRX*, *BRAF*, *CDKN2A*, *DNMT3A*, *FH*, *H3F3A*, *HRAS*, *IDH1*, *MAX*, *MEN1*, *MET*, *NF1*, *RET*, *SDHA*, *SDHAF2*, *SDHB*, *SDHC*, *SDHD*, *TMEM127*, *TP53*, and *VHL*; NGS, next generation sequencing; ***, genes investigated in NGS panel: *MAX*, *VHL*, *RET*, *NF1*, *Tp53*, *SDHD*, *SDHB*, *SDHC*, *SDHAF2*, *SDHAF3*, *SDHA*, or *TMEM127*; ****, genes investigated in NGS panel: *SDHA, SDHB, SDHC, SDHD, SDHAF2, VHL, RET, MAX, TMEM127, FH, NF1*, and *KIF1B*.

Dhanasekar K, Visakan V, Tahir F, Balasubramanian SP. Composite phaeochromocytomas-a systematic review of published literature. Langenbecks Arch Surg. 2022 Mar;407(2):517-527. doi: 10.1007/s00423-021-02129-5. Epub 2021 Mar 2. PMID: 33651160; PMCID: PMC8933353.

Arikan S, Tatar C, Emre Nayci A, Ersoz F, Baki Dogan M, Gunver F. Giant composite pheochromocytoma and gastrointestinal stromal tumor in a patient with neurofibromatosis: A case report. North Clin Istanb. 2021 Dec 29;8(6):629-633. doi: 10.14744/nci.2020.37431. PMID: 35284800; PMCID: PMC8848499.

Chen J, Wu Y, Wang P, Wu H, Tong A, Chang X. Composite pheochromocytoma/paraganglioma-ganglioneuroma: analysis of SDH and ATRX status, and identification of frequent HRAS and BRAF mutations. Endocr Connect. 2021 Aug 11;10(8):926-934. doi: 10.1530/EC-21-0300. PMID: 34261040; PMCID: PMC8428080.

Tasaka K, Ueno H, Yamasaki K, Okuno T, Isobe T , Kimura S et al Oncogenic FGFR1 mutation and amplification in common cellular origin in a composite tumor with neuroblastoma and pheochromocytoma Cancer Science (2021); 113 (4): 1535-1541

Turk Y, Karimov Z, Ozdemir M, Ertan Y, Makay O . Composite pheochromocytoma with spindle cell sarcoma- a rare tumor of the adrenal gland: case report. Hormones (Athens) (2022) march 18

Pozza C, Sesti F, Di Dato C,Sbardella E, Pofi R, Schiavi F,Bonifacio V, Isidori AM, Faggiano A,Lenzi A and Giannetta E (2020) ANovel MAX Gene Mutation Variant in aPatient With Multiple and “Composite”Neuroendocrine–NeuroblasticTumors. Front. Endocrinol. 11:234.doi: 10.3389/fendo.2020.00234

Dages KN, Kohlenberg JD, Young WF Jr, Murad MH, Prokop L, Rivera M, Dy B, Foster T, Lyden M, McKenzie T, Thompson G, Bancos I. Presentation and outcomes of adrenal ganglioneuromas: A cohort study and a systematic review of literature. Clin Endocrinol (Oxf). 2021 Jul;95(1):47-57.
